# *MCK1* is a novel regulator of *myo*-inositol phosphate synthase (MIPS) that is required for inhibition of inositol synthesis by the mood stabilizer valproate

**DOI:** 10.1371/journal.pone.0182534

**Published:** 2017-08-17

**Authors:** Wenxi Yu, Joshua Daniel, Dhara Mehta, Krishna Rao Maddipati, Miriam L. Greenberg

**Affiliations:** 1 Department of Biological Sciences, Wayne State University, Detroit, Michigan, United States of America; 2 Department of Pathology, Wayne State University, Detroit, Michigan, United States of America; Universite de Geneve, SWITZERLAND

## Abstract

*Myo*-inositol, the precursor of all inositol compounds, is essential for the viability of eukaryotes. Identifying the factors that regulate inositol homeostasis is of obvious importance to understanding cell function and the pathologies underlying neurological and metabolic resulting from perturbation of inositol metabolism. The current study identifies Mck1, a GSK3 homolog, as a novel positive regulator of inositol *de novo* synthesis in yeast. Mck1 was required for normal activity of *myo*-inositol phosphate synthase (MIPS), which catalyzes the rate-limiting step of inositol synthesis. *mck1Δ* cells exhibited a 50% decrease in MIPS activity and a decreased rate of incorporation of [^13^C_6_]glucose into [^13^C_6_]-inositol-3-phosphate and [^13^C_6_]-inositol compared to WT cells. *mck1Δ* cells also exhibited decreased growth in the presence of the inositol depleting drug valproate (VPA), which was rescued by supplementation of inositol. However, in contrast to wild type cells, which exhibited more than a 40% decrease in MIPS activity in the presence of VPA, the drug did not significantly decrease MIPS activity in *mck1Δ* cells. These findings indicate that VPA-induced MIPS inhibition is Mck1-dependent, and suggest a model that unifies two current hypotheses of the mechanism of action of VPA—inositol depletion and GSK3 inhibition.

## Introduction

*Myo*-inositol is the precursor of all inositol compounds, including phosphoinositides, inositol phosphates, inositol sphingolipids, and glycosylphosphatidylinositols. Inositol compounds are critical for nearly all cellular processes [1, 2] and are essential for the viability of eukaryotes. The pivotal role of inositol is underscored by the link between perturbation of inositol metabolism and human neurological disorders [3, 4]. Lithium, a widely used mood-stabilizing drug, was found to induce inositol depletion by inhibiting inositol monophosphatase activity [5]. Inositol depletion was thus proposed as a possible therapeutic mechanism of action of the drug [6]. Consistent with the inositol depletion hypothesis, the mood-stabilizing drug valproic acid (VPA), a short chain branched carboxylic acid with no structural similarity to lithium, was also reported to deplete intracellular inositol [7, 8]. Elucidating how inositol metabolism is regulated and how mood-stabilizing drugs deplete inositol will greatly benefit the understanding of both essential cellular functions and the pathologies underlying neurological and other illnesses associated with perturbation of inositol metabolism.

In the yeast *Saccharomyces cerevisiae*, inositol is synthesized *de novo* from glucose 6-phosphate (G-6-P) in a two-step reaction that has been well characterized. The rate-limiting enzyme *myo*-inositol-3-phosphate synthase (MIPS), which is encoded by *INO1*, converts G-6-P to inositol-3-phosphate (I-3-P) [9, 10]. In the second step, inositol-3-phosphate is dephosphorylated to *myo*-inositol by inositol monophosphatase [11, 12]. *INO1* is the most tightly controlled phospholipid biosynthesis gene. The transcription of *INO1* is regulated by the transcriptional repressor Opi1 in response to extracellular inositol levels [13]. When inositol is available in the growth medium, Opi1 translocates to the nucleus, where it represses *INO1* transcription and inhibits inositol synthesis. When inositol is limiting, Opi1 is excluded from the nucleus, and *INO1* transcription is derepressed by Ino2 and Ino4 for the *de novo* synthesis of inositol. In addition to the Ino2-Ino4-Opi1 regulatory circuit, *INO1* transcription in yeast and mammalian cells is regulated by inositol pyrophosphate synthase. Interestingly, yeast *INO1* is positively regulated by inositol pyrophosphate synthase, while mammalian cells are negatively regulated by this enzyme [14, 15]. In addition to transcriptional control of *INO1*, inositol synthesis is regulated by posttranslational modification of MIPS. Both yeast and human MIPS contain conserved phosphorylation sites, which regulate the enzymatic activity of MIPS [16, 17]. Inositol synthesis is thus highly regulated in both yeast and mammalian cells by multiple mechanisms at the level of *INO1*.

In light of the key role of MIPS in the regulation of inositol synthesis, elucidating the mechanism whereby MIPS is affected by the inositol-depleting drug VPA is of obvious importance. Yeast cells treated with therapeutically relevant concentrations of VPA exhibited decreased levels of inositol-3-phosphate and inositol, consistent with decreased MIPS activity [7]. Both yeast and human MIPS purified from yeast cells grown in the presence of VPA exhibited decreased enzymatic activity, consistent with covalent modification of the enzyme [17, 18]. However, *in vitro* activity of purified yeast or mammalian MIPS was not affected by VPA, indicating that inhibition is indirect [8, 18]. Interestingly, mutation of two phosphorylation sites in MIPS decreased VPA-induced MIPS inhibition [17], suggesting that inhibition of the enzyme by VPA may be mediated by a phosphorylation cascade.

While the inositol depletion hypothesis has stimulated considerable research into the mechanisms of action of mood stabilizing drugs, inhibition of GSK3 has also been proposed as a therapeutic mechanism of action. Lithium was first reported to cause GSK3β inhibition in *Xenopus* [19]. Inhibition of GSK3 by lithium was also observed in other model systems [20–24]. Interestingly, VPA has also been shown to inhibit GSK3 in several studies [25–28], suggesting that GSK3 inhibition may account for the therapeutic effect of mood-stabilizing drugs. GSK3 is a serine/threonine kinase that exerts regulatory functions in many cellular events [29–31]. GSK3α and GSK3β, which contain highly conserved amino acid sequences in their kinase domains, are two major GSK3 isoforms expressed in mammalian cells [32, 33]. GSK3β is predominant expressed in the brain. The alteration of GSK3β-mediated signaling pathways is associated with neuronal disorders and cancer [30, 31, 34, 35]. Consistent with the GSK3 inhibition hypothesis, GSK3 inhibitors exhibited mood-stabilizing effects in animal studies. In rodents, GSK3 inhibitors AR-A014418 and L803-mts exhibited anti-depressive effects in forced swim tests [36–38]. GSK3 inhibitors also antagonized amphetamine-induced hyperactivity, a rodent model of mania [37, 39]. Therefore, similar to inositol depletion, GSK3 inhibition is a common outcome of VPA and lithium and is associated with mood-stabilizing effects.

Although the inositol depletion and GSK3 inhibition hypotheses of mood stabilization have been suggested independently based on seemingly unrelated studies, our findings suggest that they may be linked by a common mechanism [40]. In light of published findings that VPA causes inositol depletion by indirectly inhibiting MIPS [18], that VPA inhibits GSK3 (23–26), and that yeast cells lacking all four GSK3 homologs (*MCK1*, *MDS1*, *MRK1* and *YGK3*) exhibit inositol depletion [41], we hypothesized that VPA inhibits MIPS by a mechanism involving negative regulation of one or more GSK3 homologs, thereby causing inositol depletion. In this report, we demonstrate for the first time that the GSK-3 homolog *MCK1* (but not *MDS1*, *MRK1* or *YGK3*) is required for the optimal synthesis of inositol. Importantly, VPA does not inhibit MIPS in *mck1*Δ cells, indicating that Mck1 is a downstream target of VPA in MIPS inhibition.

## Materials and methods

### Yeast strains

All strains used in this study were in the W303 background. The WT strain was crossed with the isogenic *gsk3Δ* (*mck1Δmrk1Δmds1Δygk3Δ*) mutant for the generation of haploid spores, from which isogenic *mck1Δ*, *mrk1Δ*, *mds1Δ* and *ygk3Δ* strains were derived. The *GSK3* genotype of each mutant strain was confirmed by PCR. For determination of MIPS enzymatic activity, yeast strains that harbor the His-Xpress tagged MIPS gene at the *INO1* locus were constructed. To do so, the *INO1* gene was first replaced by a KanMX cassette, which was subsequently replaced by an N-terminal His-Xpress tagged *INO1* cassette cloned from the pRD-INO1 plasmid [17].

### Growth media

Yeast cells were grown at 30°C, 37°C or 38°C in synthetic complete (SC) medium, which contained glucose (2% w/v), adenine (20 mg/liter), arginine (20 mg/liter), histidine (20 mg/liter), methionine (20 mg/liter), tryptophan (20 mg/liter), leucine (60 mg/liter), lysine (200 mg/liter), threonine (300 mg/liter), ammonium sulfate (0.2% w/v), inositol-free Difco vitamin mix, vitamin-free yeast base, plus agar (2% w/v) for solid medium. Inositol (75 μM) and VPA (1 mM) were added separately as indicated.

### Measurement of intracellular inositol levels

Intracellular inositol levels were determined using the method of Maslanski and Busa with modification [42]. Briefly, cells were lysed in dH_2_O containing 1X protease inhibitor by vortexing with acid-washed glass beads at 4°C. Cell extracts were mixed with 7.5% perchloric acid and centrifuged at 10,000 g for 10 min at 4°C. Supernatants were collected and titrated with ice cold KOH to pH 7. Samples were clarified by centrifugation and loaded onto columns containing 1 ml AG 1-X8 resin/H_2_0 (1:1) mixture. Inositol was eluted with 5 ml dH_2_O, and eluates were dried in an oven at 70°C and stored at -80°C. Prior to assay, samples were dissolved in dH_2_O. Inositol content in samples was measured as described previously [43].

### *In vivo* assay of the rate of inositol *de novo* synthesis

Cells were grown in SC I^+^ medium to the mid log phase, washed twice with dH_2_O, transferred to SC I^-^ medium, and incubated for 1 h or 3 h. [^13^C_6_]-glucose was added to a final concentration of 0.2%. After 15 min, cells were harvested and lysed in dH_2_O containing 1X protease inhibitor by vortexing with acid-washed glass beads at 4°C. Cell extracts were mixed with acetonitrile to a final concentration of 90% and centrifuged at 16,000 g for 10 min at 4°C to remove precipitated protein. Supernatants were collected and stored at -20°C for the analysis of ^13^C labeled inositol-3-phosphate ([^13^C_6_]-I-3-P) and inositol ([^13^C_6_]-Inositol) by LC-MS. [^13^C_6_]-I-3-P and [^13^C_6_]-Inositol were quantified by LC-MS as follows: Samples were subjected to HPLC on a Diamond Hydride column (Cogent HPLC Columns, Microsolv Technology Corporation, 2 mm x 150 mm, 4μ, 100 Å) using gradient elution. The gradient between mobile phases A: acetonitrile-aqueous ammonium formate (9:1 v/v) and B: acetonitrile-0.1% aqueous formic acid (1:9 v/v) was: 0 min– 95% A, 2 min– 80% A, 4 min– 30%A, 7 min– 30%A. At the end of each chromatographic run, the mobile phase composition was changed to initial conditions and equilibrated for 5 min before the start of the next sample analysis. The flow rate was 0.2 ml/min and the column was maintained at 35°C. The eluent was directly introduced to the mass analyzer (QTRAP5500, Sciex) via a TurboIon Electrospray ionization source. The following parameters were used for the mass analyzer: Ion spray voltage: -4500 V, Curtain gas: 35, Source temperature: 600°C, GS1 & GS2: 45, and EP & CXP: 10. [^13^C_6_]-I-3-P and [^13^C_6_]-inositol were monitored by Multiple Reaction Monitoring with the following transitions, declustering potentials (DP), and collision energies (CE). [^13^C_6_]-I-3-P *m/z* 265.1 to 79 at DP: -110 V and CE: -25 V; [^13^C_6_]-inositol *m/z* 185.1 to 89 at DP: -96 V and CE: -21 V. Under these conditions, the retention times for [^13^C_6_]-I-3-P and [^13^C_6_]-inositol were 4.7 and 3.6 min, respectively. The detection limits were 0.1 ng and the quantitation limits were 0.4 ng on the column for each analyte.

### MIPS activity assay

Cells expressing His-Xpress tagged MIPS were grown to the early stationary phase and lysed in buffer (50 mM Tris-HCl pH 7.5, 0.6 M sorbitol, 0.3 M NaCl, 1X protease inhibitor and 1X phosphatase inhibitor) by vortexing with acid-washed glass beads at 4°C. MIPS protein was purified from cell extracts using the PureProteome^™^ Nickel Magnetic Bead System (Millipore). Purified MIPS protein was dialyzed (1 mM Tris acetate pH 8.0, 0.05 M dithiothreitol, 0.025X protease inhibitor and 0.1X phosphatase inhibitor) and concentrated with an Amicon Ultra-0.5 Centrifugal Filter (Millipore). MIPS protein concentration was determined by Bradford assay. Enzymatic activity of 3 μg purified MIPS was determined by enzyme-coupled colorimetric assay [44].

### Immunoblotting

Cell extract was obtained as described above and protein concentration was determined by the Bradford assay (Bio-Rad). Extract corresponding to 30–40 μg protein was analyzed by SDS-PAGE on a 10% gel. Immunoblotting was performed using primary antibodies against the Xpress tag (R901-25, mouse, Thermo Fisher), β-actin (mAbGEa, mouse, Thermo Fisher), followed by corresponding secondary antibodies.

## Results

### Deletion of *MCK1* leads to inositol depletion

**Fig 1:**
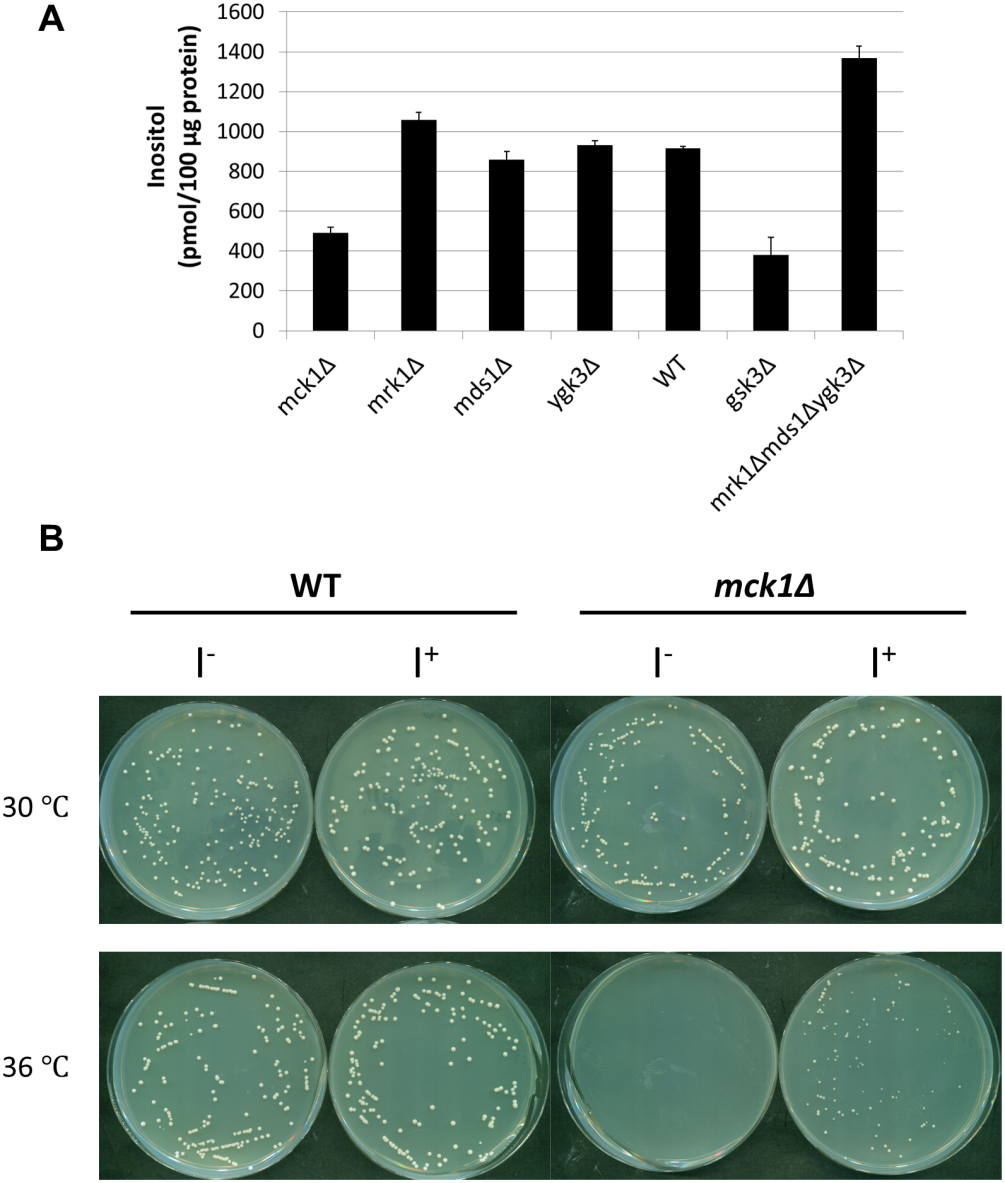
Decreased intracellular inositol levels in *mck1Δ* cells. (A) Cells were grown in SC medium to the early stationary phase. Intracellular inositol levels were determined as described in “*Experimental procedures*” (3 independent experiments with 3 replicates each). Values shown are mean ± SEM. (B) Inositol restored the growth of *mck1*Δ cells at elevated temperature. WT and *mck1*Δ cells were diluted and plated on SC plates in the presence or absence of 75 M inositol and incubated at indicated temperatures for 2 days.

We have previously shown that yeast cells lacking all four GSK3 homologs (*gsk3Δ*) exhibit decreased inositol synthesis [45]. To identify the *GSK3* gene required for inositol synthesis, isogenic mutants were constructed with all combinations of gsk3 mutations. As seen in Fig 1A, a nearly 50% drop in intracellular inositol levels was observed in *mck1Δ* cells, similar to the 60% decrease in inositol levels seen in *gsk3Δ* cells. The presence of only the *MCK1* gene (i.e., triple mutant *mrk1Δmds1Δygk3Δ*) was sufficient for optimal inositol synthesis. Consistent with decreased intracellular inositol levels, *mck1*Δ cells exhibited decreased growth at 36°C, which was rescued by supplementation of inositol (Fig 1B). These findings indicate that Mck1 is a positive regulator of inositol synthesis.

### Mck1 regulates the rate of inositol *de novo* synthesis

**Fig 2:**
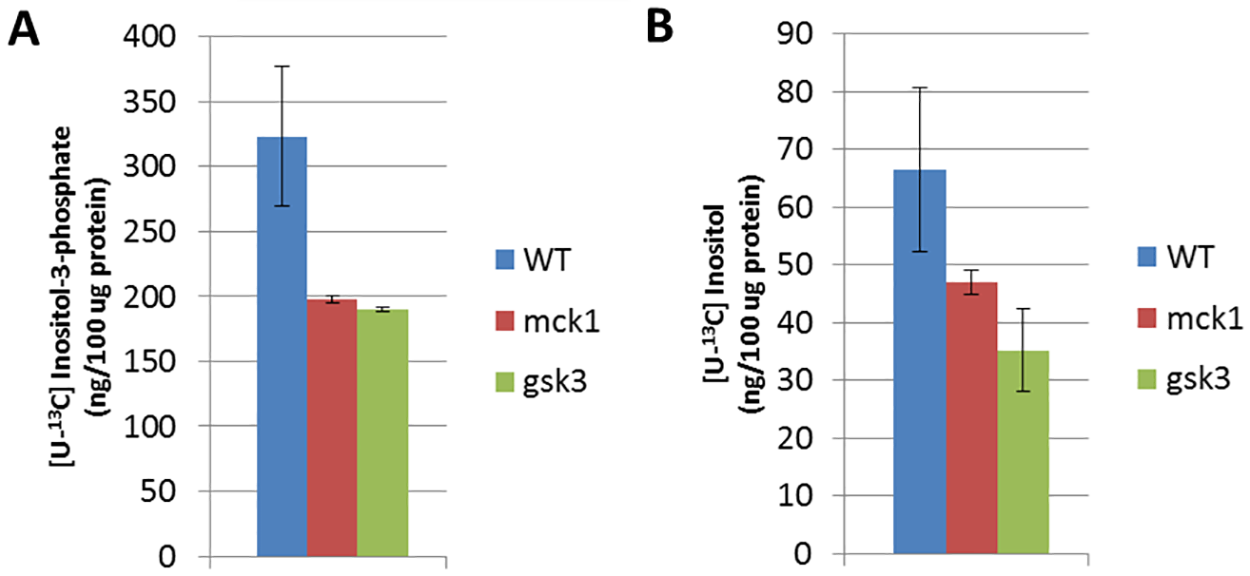
Rate of synthesis of inositol-3-phosphate and inositol is decreased in *mck1Δ* cells. Cells were cultured in SC medium supplemented with 75 M inositol to the mid log phase and transferred to SC inositol free medium for 3 h. [^13^C_6_]glucose was added at a final concentration of 0.2% and cells were incubated for 15 min. Levels of ^13^C labeled inositol-3-phosphate (A) and inositol (B) in cell extracts were determined by LC-MS (6 independent experiments). Values shown are mean ± SEM.

We wished to determine if decreased intracellular inositol levels in *mck1Δ* cells reflected a decreased rate of synthesis of inositol *in vivo*. To do so, cells were grown in I^+^ medium, washed, and transferred to I^-^ medium for 3 h. [^13^C_6_]glucose was then added, and synthesis of ^13^C labeled inositol-3-phosphate and inositol (designated as [^13^C_6_]-I-3-P and [^13^C_6_]-inositol, respectively) was detected by LC-MS. As seen in Fig 2, *mck1Δ* and *gsk3Δ* cells exhibited significantly lower levels of [^13^C_6_]-I-3-P and [^13^C_6_]-inositol compared to WT cells. These results indicate that Mck1 is required for optimal *de novo* synthesis of inositol.

### Mck1 regulates MIPS activity

**Fig 3:**
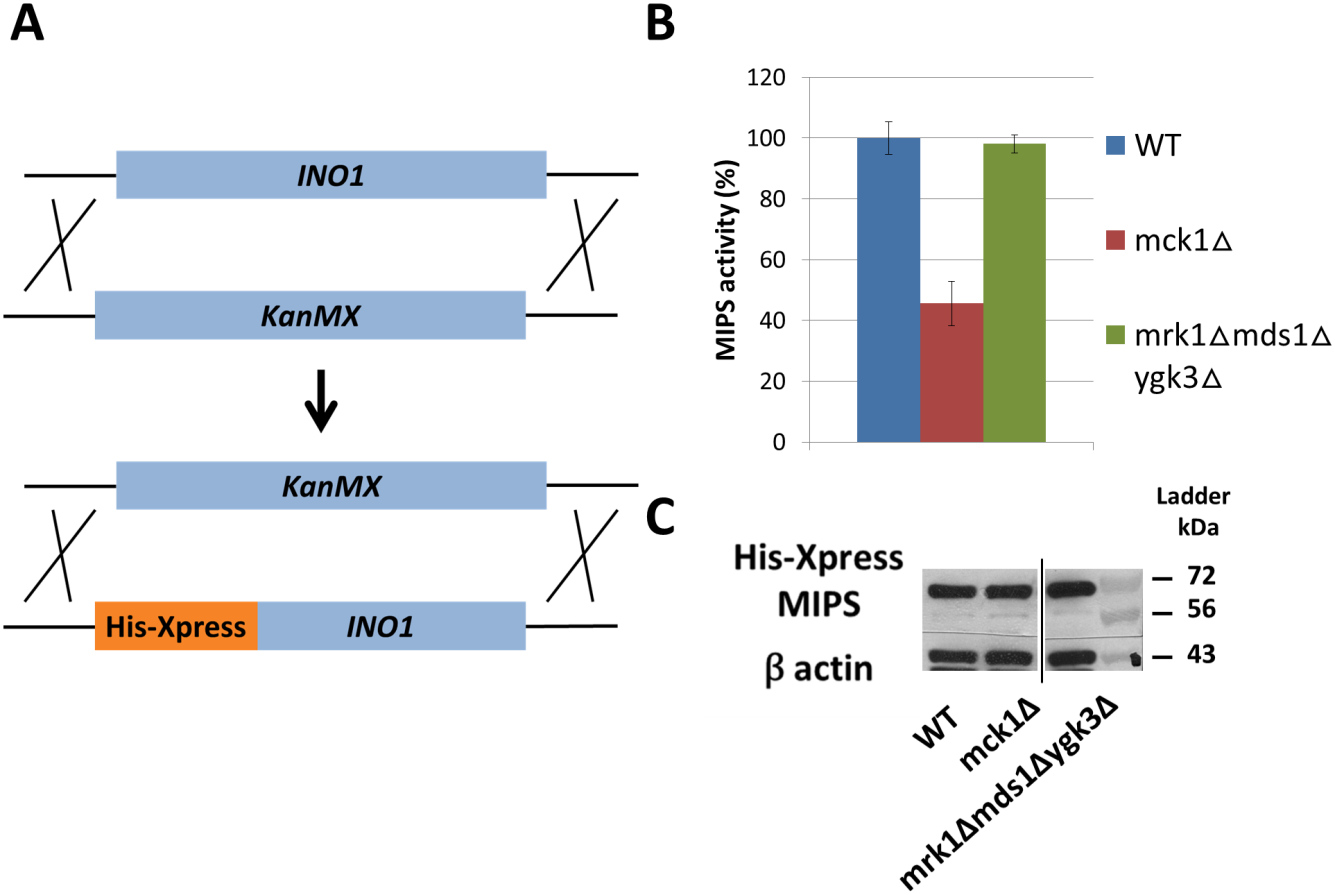
MIPS activity is decreased in *mck1Δ* cells. (A) Strains expressing His-Xpress tagged MIPS were constructed by knocking out the *INO1* gene with the *KanMX* gene, which is then replaced with the *His-Xpress INO1* fusion gene. (B) Cells expressing the His-Xpress tagged MIPS were grown in SC medium to the early stationary phase. Enzymatic activity of MIPS protein purified from cell extracts was determined as described in “*Experimental procedures*” (2 independent experiments with 3 replicates each). Values shown are mean ± SEM. (C) MIPS protein levels are not changed in *gsk3* mutant cells. 30 g protein from total cell extract were resolved with 10% SDS-PAGE and analyzed by Western blot.

MIPS catalyzes the rate-limiting step of inositol *de novo* synthesis. To ascertain if decreased inositol synthesis in *mck1Δ* cells resulted from decreased enzymatic activity of MIPS, isogenic strains expressing His-Xpress tagged MIPS in the genomic site were constructed (Fig 3A). Compared to WT, a 50% decrease in activity was observed in MIPS purified from *mck1Δ* cells (Fig 3B), consistent with the loss of a positive regulator of MIPS in the mutant. MIPS activity in the *mrk1Δmds1Δygk3Δ* triple mutant was similar to that of WT, in agreement with the finding of WT inositol levels in the mutant (Fig 1A). WT and mutant cells contained similar levels of MIPS protein (Fig 3C), indicating that the deletion of *MCK1* does not affect MIPS synthesis. Taken together, these findings indicate that deletion of *MCK1* results in inositol depletion due to decreased activity of MIPS.

### VPA-induced MIPS inhibition is Mck1 dependent

**Fig 4:**
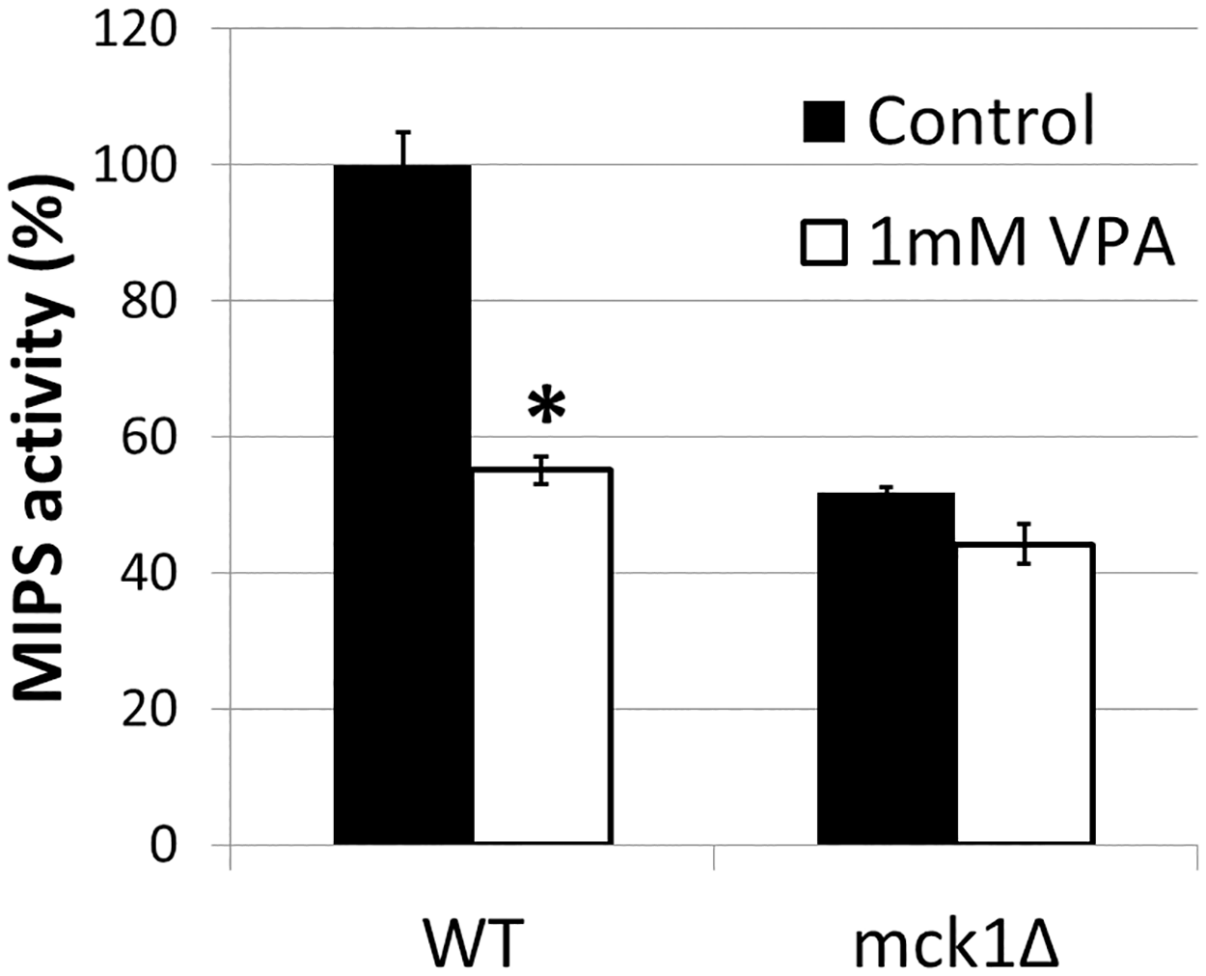
VPA does not decrease MIPS activity in *mck1Δ* cells. Cells expressing His-Xpress tagged MIPS were grown in SC medium to the mid log phase and treated with 1 mM VPA for 3 h. MIPS activities were determined as described in “*Experimental procedures*” (3 independent experiments with 3 replicates each) (*p<0.05). Values shown are mean ± SEM. Statistical significance was determined by unpaired *t* test.

In light of our previous reports that VPA indirectly inhibits MIPS [8, 18], our current finding that Mck1 is a positive regulator of MIPS activity suggests a mechanism in which VPA causes inositol depletion by direct or indirect inhibition of Mck1. If VPA inhibition of MIPS activity were mediated by Mck1, then VPA would not cause a further decrease in MIPS activity in the *mck1Δ* mutant. To address this possibility, MIPS activity was measured in *mck1Δ* cells grown in the presence or absence of VPA. As shown in Fig 4, WT cells treated with VPA exhibited more than a 40% decrease in MIPS activity. However, VPA did not significantly decrease MIPS activity in *mck1Δ* cells. These findings suggest that the effects of loss of Mck1 and VPA treatment are not additive, and that VPA-induced MIPS inhibition is Mck1-dependent.

## Discussion

The current study demonstrates for the first time that *de novo* synthesis of inositol is positively regulated by Mck1, which is required for normal activity of the enzyme (MIPS) catalyzing the rate-limiting step of inositol synthesis. Our findings also identify Mck1 as a possible downstream target of VPA.

Among mutants of the four GSK3 homologs, *mck1Δ*, *mrk1Δ*, *mds1Δ*, and *ygk3Δ*, only *mck1Δ* cells exhibited decreased intracellular inositol levels, as well as inositol dependent growth at elevated temperature (Fig 1). The *mrk1Δmds1Δygk3Δ* triple mutant containing only the wild type *MCK1* homolog had increased intracellular inositol levels (Fig 1B). These findings indicate that *MCK1* is the GSK3 homolog that is necessary and sufficient for normal intracellular inositol levels. Somewhat increased levels of inositol in *mrk1Δmds1Δygk3Δ* cells suggests that one or more of these genes may negatively regulate inositol synthesis.

MIPS purified from *mck1Δ* cells exhibited WT levels of MIPS protein but decreased enzymatic activity (Fig 3), suggesting that *MCK1* affects enzymatic activity, but not synthesis, of MIPS. Consistent with decreased MIPS activity, the rate of inositol synthesis was decreased in *mck1Δ* cells (Fig 2). These findings strongly support a regulatory role of Mck1 in controlling inositol synthesis. We have previously shown that MIPS is a phosphoprotein with at least three potential phosphosites. While Mck1 has GSK3 kinase enzymatic activity, it does not directly phosphorylate MIPS (data not shown). However, the possibility remains that Mck1 indirectly affects MIPS activity by activating a phosphorylation cascade that phosphorylates the enzyme.

As both *MCK1* ablation and VPA lead to similar cellular effects, including inositol depletion [7, 8] and MIPS inhibition (Fig 4), we considered the possibility that they are components of a single mechanism. In support of this, VPA did not significantly decrease MIPS activity in *mck1Δ* cells (Fig 4). This finding suggests that Mck1 acts downstream of VPA to regulate MIPS. We have previously shown that VPA causes increased phosphorylation of MIPS (17). It is possible that VPA affects MIPS phosphorylation via an Mck1 phosphorylation cascade.

**Fig 5:**
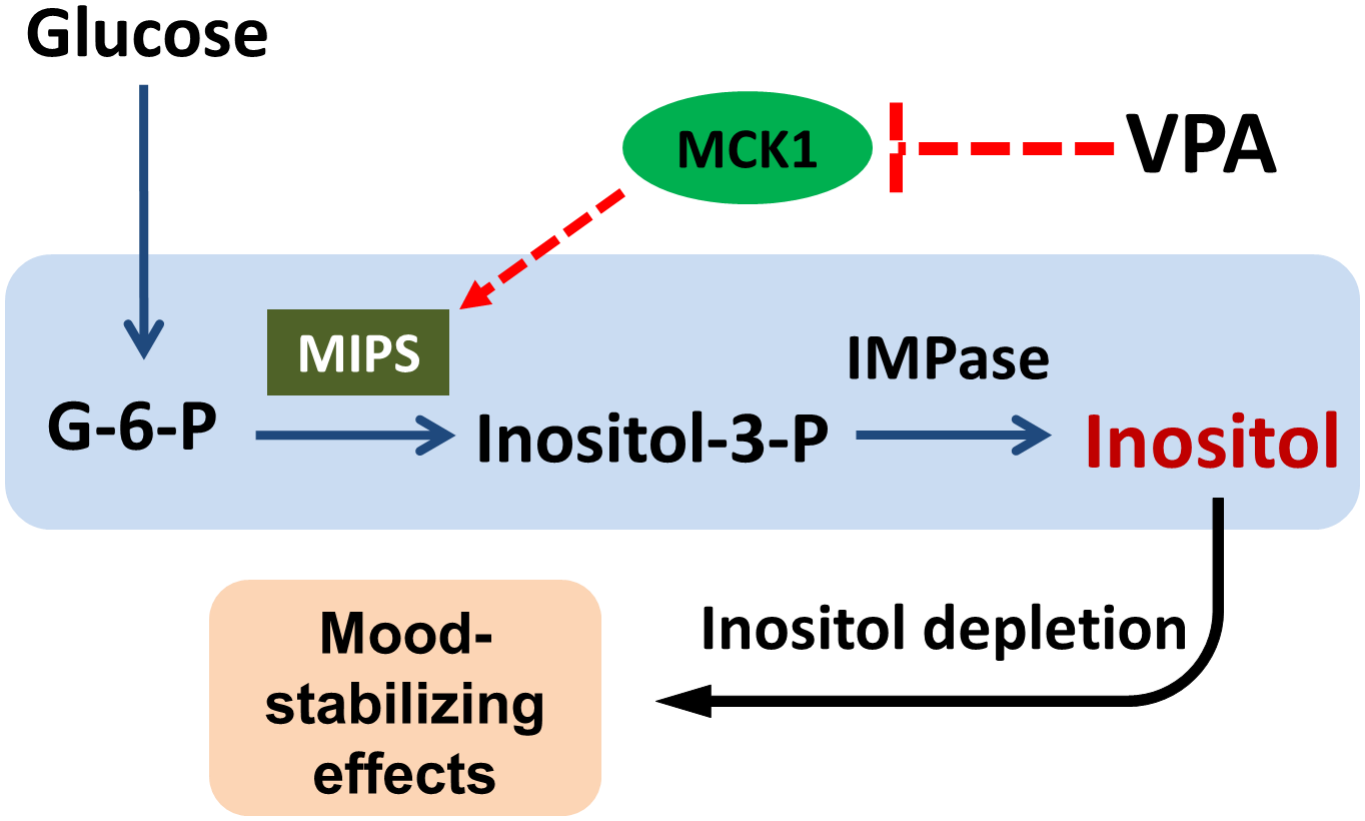
Model of VPA-induced inositol depletion via inhibition of Mck1. MIPS is the rate-limiting enzyme of inositol synthesis. Mck1 is a positive regulator of MIPS that is required for optimal *de novo* synthesis of inositol. Positive regulation of MIPS by Mck1 is inhibited by VPA, thus accounting for VPA-induced inhibition of MIPS and concomitant decrease in inositol synthesis. Inositol depletion causes a plethora of cellular consequences, including those that contribute to mood-stabilization.

This study demonstrates for the first time that Mck1 is required for VPA-mediated MIPS inhibition, and for optimal inositol synthesis *in vivo*. These findings have implications for understanding the mechanisms that control inositol synthesis and VPA-induced inositol depletion, and suggest that two common outcomes in published reports of VPA effects, inositol depletion and GSK3 inhibition, are intrinsically related. We suggest that inositol depletion and GSK3 inhibition may be mechanistically inter-related (Fig 5). Accordingly, inositol depletion results from decreased MIPS activity caused by VPA induced inhibition of Mck1/GSK3. Inositol-containing molecules are involved in the regulation of growth, protein secretion, apoptosis, and many other cellular events [3, 46, 47]. The alteration of inositol homeostasis affects hundreds of signaling pathways and leads to perturbation of numerous cellular functions [48, 49], among which are those that are related to the mood-stabilizing effects of VPA.

## Abbreviations

[^13^C_6_]-I-3-P: ^13^C labeled inositol-3-phoshpate
[^13^C_6_]-Inositol: ^13^C labeled inositol
G-6-P: glucose 6-phosphate
I-3-P: inositol-3-phosphate
MIPS: *myo*-inositol-3-phosphate synthase
VPA: valproic acid
